# Supplementary material for: Effectiveness of psychological crisis interventions during infectious disease outbreaks in low- and middle-income countries: a systematic review of Randomized Control Trials
Source: Glob Ment Health (Camb). 2025 Feb 26;12:e32. doi: 10.1017/gmh.2025.22 (PMC12037349; doi:10.1017/gmh.2025.22)
Supplement: Yirdaw et al. supplementary material [file S2054425125000226sup001.docx]

**Search strategy**

**Keywords and Mesh terms**

**Psychological interventions**

“psychological treatment"; “psychological intervention”; "psychological support*"; "psychosocial treatment*" ; "psychosocial intervention*” ; "psychosocial support*"; “psychoeducation*” ; “counsel*”; "cognitive behavioural therap*” ; "acceptance? commitment therap*" ; "cognitive analytic therap*"; "family therap*" ; "group therapy*" ; "interpersonal therapy*" ; "interpersonal psychotherap*" ; "problem solving therap*"; "problem solving psychotherap*" ; "mindfulness therap*" ; "motivational interview*" ; "psychodynamic psychotherap*" ; "supportive psychotherap*" ; "relaxation therap*" ; "rational-emotive psychotherap*" ; "Psychotherapy" ; "Psychotherapy, Psychodynamic" ; "Imagery, Psychotherapy" ; "Psychotherapy, Rational-Emotive" ; "Psychotherapy, Multiple" ; "Psychotherapy, Group" ; "Psychotherapy, Brief" ; "Person-Centered Psychotherapy" ; "Interpersonal Psychotherapy" ; "Cognitive Behavioral Therapy" ; "Crisis Intervention"

**Outbreaks**

“disease outbreak"; Epidemic*; Pandemic*; epidemic*; "disease Epidemic"; coronavir*; "corona virus"

"corona pandemic"; betacoronavir*; covid19)) ; covid; nCoV; "novel CoV"; "CoV 2"; CoV2; sarscov2; sars2

2019nCoV; "wuhan virus"; Sars; “severe acute respiratory ; pneumonia*; outbreak*; "coronavirus Infection"; Coronavirus; Betacoronavirus; MERS-CoV; "Middle East Respiratory Syndrome"; Ebolavirus; Ebola; "Ebola virus"; "Ebola Virus Disease"; "Ebola Hemorrhagic Fever"; "dengue virus"; "encephalitis viruses"; "encephalitis viruses"; "yellow fever virus" ; "zika virus"; Listeriosis; Meningitis; "Chikungunya Fever"; "Yellow fever"; Measles

**List of low and middle-income countries (LMICS)**

**PubMed**

((((((((((((((((((((((((("psychological treatment*"[46]) OR ("psychological intervention*"[46])) OR ("psychological support*"[46])) OR ("psychosocial treatment*"[46])) OR ("psychosocial intervention*"[46])) OR ("psychosocial support*"[46])) OR (psychoeducation*[46])) OR (counsel*[46])) OR ("cognitive behavioural therap*"[46])) OR ("acceptance* commitment therap*"[46])) OR ("cognitive analytic therap*"[46])) OR ("family therap*"[46])) OR ("group therapy*"[46])) OR ("interpersonal therapy*"[46])) OR ("interpersonal psychotherap*"[46])) OR ("problem solving therap*"[46])) OR ("problem solving psychotherap*"[46])) OR ("mindfulness therap*"[46])) OR ("motivational interview*"[46])) OR ("psychodynamic psychotherap*"[46])) OR ("supportive psychotherap*"[46])) OR ("relaxation therap*"[46])) OR ("rational-emotive psychotherap*"[46])) OR (("Psychotherapy"[47] OR "Psychotherapy, Psychodynamic"[47] OR "Imagery, Psychotherapy"[47] OR "Psychotherapy, Rational-Emotive"[47] OR "Psychotherapy, Multiple"[47] OR "Psychotherapy, Group"[47] OR "Psychotherapy, Brief"[47] OR "Person-Centered Psychotherapy"[47] OR "Interpersonal Psychotherapy"[47] OR "Cognitive Behavioral Therapy"[47]) OR "Crisis Intervention"[47])) AND ((((((((((((((((((((((((((((((((((((((((((((("disease outbreak") OR (epidemic*)) OR (pandemic*)) OR (epidemic*)) OR ("disease Epidemic")) OR (coronavir*)) OR ("corona virus")) OR ("corona pandemic")) OR (betacoronavir*)) OR (covid19)) OR (covid)) OR (nCoV)) OR ("novel CoV")) OR ("CoV 2")) OR (CoV2)) OR (sarscov2)) OR (sars2)) OR (2019nCoV)) OR ("wuhan virus")) OR (sars)) OR (severe acute respiratory OR pneumonia*)) OR (outbreak*)) OR ("coronavirus Infection")) OR (coronavirus)) OR (betacoronavirus)) OR (MERS-CoV)) OR ("Middle East Respiratory Syndrome")) OR (ebolavirus)) OR (Ebola)) OR ("Ebola virus")) OR ("Ebola Virus Disease")) OR ("Ebola Hemorrhagic Fever")) OR ("hiv infection")) OR ("acquired immunodeficiency syndrome")) OR ("acute retroviral syndrome")) OR ("dengue virus")) OR ("encephalitis viruses")) OR ("encephalitis viruses")) OR ("yellow fever virus")) OR ("zika virus")) OR (listeriosis)) OR (meningitis)) OR ("Chikungunya Fever")) OR ("Yellow fever")) OR (measles))) AND ("Developing Countries"[47] OR developing countr*[48] OR developing nation*[48] OR less developed countr*[48] OR less developed nation*[48] OR third world nation*[48] OR third world countr*[48] OR under developed nation*[48] OR underdeveloped nation*[48] OR under developed countr*[48] OR underdeveloped nation*[48] OR middle income countr*[48] OR middle income nation*[48] OR low income countr*[48] OR low income nation*[48] OR poor countr*[48] OR poor nation*[48] OR lmic[48] OR lmics[48] OR "Africa"[47] OR "Asia"[47] OR "South America"[47] OR "Latin America"[47] OR "Central America"[47] OR africa[48] OR asia[48] OR south america[48] OR latin america[48] OR central america[48] OR Afghanistan*[48] OR Albania*[48] OR Algeria*[48] OR Samoa*[48] OR Angola*[48] OR Armenia*[48] OR Azerbaijan*[48] OR Bangladesh*[48] OR Bengali[48] OR Belarus*[48] OR Belize[48] OR Benin[48] OR Bhutan*[48] OR Bolivia*[48] OR Bosnia*[48] OR Herzegovina*[48] OR Botswana*[48] OR Brazil*[48] OR Bulgaria*[48] OR Burkina Faso[48] OR Burundi*[48] OR Cabo Verd*[48] OR Cape Verd*[48] OR Cambodia*[48] OR Cameroon*[48] OR Central African*[48] OR Chad*[48] OR China[48] OR Chinese OR Colombia*[tiab] OR Comoros[tiab] OR Congo[tiab] OR Costa Rica*[tiab] OR Cote d'Ivoire[tiab] OR Ivory Coast[tiab] OR Cuba[tiab] OR Cuban[tiab] OR Djibouti[tiab] OR Dominica* [tiab] OR Ecuador[tiab] OR Egypt[tiab] OR El Salvador*[tiab] OR Eritrea*[tiab] OR Ethiopia*[tiab] OR Fiji*[tiab] OR Gabon*[tiab] OR Gambia*[tiab] OR Georgia*[tiab] OR Ghana*[tiab] OR Grenada*[tiab] OR Guatemala*[tiab] OR Guinea*[tiab] OR Guyan*[tiab] OR Haiti*[tiab] OR Hondura*[tiab] OR India[tiab] OR Indian*[tiab] OR Indonesia*[tiab] OR Iran*[tiab] OR Iraq*[tiab] OR Jamaica*[tiab] OR Jordan*[tiab] OR Kazakh*[tiab] OR Kenya*[tiab] OR Kiribati[tiab] OR People's Republic of Korea[tiab] OR North Korea[tiab] OR Kosovo[tiab] OR Kosovar* [tiab] OR Kyrgyz*[tiab] OR Lao[tiab] OR Laos[tiab] OR Laotian*[tiab] OR Lebanon[tiab] OR Lebanes*[tiab] OR Lesotho[tiab] OR Liberia*[tiab] OR Libya*[tiab] OR Macedonia*[tiab] OR Madagascar*[tiab] OR Malawi*[tiab] OR Malaysia*[tiab] OR Maldives[tiab] OR Mali[tiab] OR Marshall Island*[tiab] OR Mauritania*[tiab] OR Mauriti*[tiab] OR Mexico[tiab] OR Mexican*[tiab] OR Micronesia*[tiab] OR Moldova*[tiab] OR Mongolia*[tiab] OR Montenegr*[tiab] OR Morocc*[tiab] OR Mozambique[tiab] OR Myanmar[tiab] OR Burmese*[tiab] OR Burma[tiab] OR Namibia*[tiab] OR Nepal*[tiab] OR Nicaragua*[tiab] OR Niger*[tiab] OR Pakistan*[tiab] OR Palau[tiab] OR Panama*[tiab] OR Paraguay*[tiab] OR Peru*[tiab] OR Philippin*[tiab] OR Romania*[tiab] OR Rwanda*[tiab] OR Samoa*[tiab] OR Sao Tome[tiab] OR Principe[tiab] OR Senegal*[tiab] OR Serbia*[tiab] OR Sierra Leone*[tiab] OR Solomon Island*[tiab] OR Somalia* [tiab] OR South Africa*[tiab] OR Sri Lanka[tiab] OR St Lucia[tiab] OR Saint Lucia[tiab] OR St Vincent[tiab] OR Saint Vincent[tiab] OR Grenadines[tiab] OR Sudan*[tiab] OR Suriname*[tiab] OR Swaziland*[tiab] OR Syria*[tiab] OR Tajik*[tiab] OR Tanzania*[tiab] OR Thai*[tiab] OR Timor*[tiab] OR Togo*[tiab] OR Tonga*[tiab] OR Tunisia*[tiab] OR Turkey[tiab] OR Turkish[tiab] OR Turkmen*[tiab] OR Tuvalu*[tiab] OR Uganda*[tiab] OR Ukrain*[tiab] OR Uzbeki*[tiab] OR Vanuatu*[tiab] OR Vietnam*[tiab] OR Viet nam*[tiab] OR West Bank[tiab] OR Gaza*[tiab] OR Palestin*[tiab] OR Yemen*[tiab] OR Zambia*[tiab] OR Zimbabw*[tiab])

**Scopus**

( TITLE-ABS-KEY ( ( outbreak* OR epidemic* OR pandemic* OR epidemic* OR coronavir* OR "corona virus" OR "corona pandemic" OR covid19 OR covid OR "novel CoV" OR sarscov2 OR 2019ncov OR "wuhan virus" OR sars OR "severe acute respiratory" OR "coronavirus Infection" OR coronavirus OR mers-cov OR "Middle East Respiratory Syndrome" OR ebolavirus OR ebola OR "Ebola virus" OR "Ebola Virus Disease" OR "dengue virus" OR "yellow fever virus" OR "zika virus" OR "Chikungunya Fever" OR "Yellow fever" OR measles ) ) ) AND ( TITLE-ABS-KEY ( "psychological treatment" OR "psychological intervention" OR "psychological support" OR "psychosocial treatment" OR "psychosocial intervention" OR "psychosocial support" OR psychoeducation OR counsel* OR "cognitive behavioural therap" OR "acceptance commitment therap" OR "cognitive analytic therapy" OR "family therapy" OR "group therapy" OR "problem solving therap" OR "problem solving psychotherapy" OR "mindfulness therapy" OR "motivational interview" OR "supportive psychotherapy" OR "relaxation therapy" OR "Psychodynamic psychotherapy" OR "Imagery Psychotherapy" OR "Rational-Emotive" OR psychotherapy OR "Brief Psychotherapy" OR "Person Centered Psychotherapy" OR "Interpersonal Psychotherapy" OR "Cognitive Behavioral Therapy" OR "Crisis Intervention" ) ) AND ( LIMIT-TO ( SUBJAREA , "PSYC" ) OR LIMIT-TO ( SUBJAREA , "SOCI" ) OR LIMIT-TO ( SUBJAREA , "MEDI" ) ) AND ( LIMIT-TO ( DOCTYPE , "ar" ) ) AND ( LIMIT-TO ( LANGUAGE , "English" ) ) AND ( LIMIT-TO ( EXACTKEYWORD , "Article" ) OR LIMIT-TO ( EXACTKEYWORD , "Adult" ) OR LIMIT-TO ( EXACTKEYWORD , "COVID-19" ) OR LIMIT-TO ( EXACTKEYWORD , "Pandemic" ) OR LIMIT-TO ( EXACTKEYWORD , "Coronavirus Disease 2019" ) OR LIMIT-TO ( EXACTKEYWORD , "Pandemics" ) OR LIMIT-TO ( EXACTKEYWORD , "Mental Health" ) OR LIMIT-TO ( EXACTKEYWORD , "Depression" ) OR LIMIT-TO ( EXACTKEYWORD , "Anxiety" ) OR LIMIT-TO ( EXACTKEYWORD , "Counseling" ) OR LIMIT-TO ( EXACTKEYWORD , "Epidemic" ) OR LIMIT-TO ( EXACTKEYWORD , "Psychotherapy" ) OR LIMIT-TO ( EXACTKEYWORD , "Young Adult" ) OR LIMIT-TO ( EXACTKEYWORD , "Patient Counseling" ) OR LIMIT-TO ( EXACTKEYWORD , "Telemedicine" ) OR LIMIT-TO ( EXACTKEYWORD , "Cognitive Behavioral Therapy" ) OR LIMIT-TO ( EXACTKEYWORD , "Social Support" ) OR LIMIT-TO ( EXACTKEYWORD , "Anxiety Disorder" ) OR LIMIT-TO ( EXACTKEYWORD , "Telehealth" ) OR LIMIT-TO ( EXACTKEYWORD , "Randomized Controlled Trial" ) OR LIMIT-TO ( EXACTKEYWORD , "Public Health" ) OR LIMIT-TO ( EXACTKEYWORD , "Mental Stress" ) OR LIMIT-TO ( EXACTKEYWORD , "Disease Severity" ) OR LIMIT-TO ( EXACTKEYWORD , "Coronavirus Infection" ) OR LIMIT-TO ( EXACTKEYWORD , "Coronavirus Infections" ) OR LIMIT-TO ( EXACTKEYWORD , "Prevention And Control" ) OR LIMIT-TO ( EXACTKEYWORD , "Treatment Outcome" ) OR LIMIT-TO ( EXACTKEYWORD , "Stress" ) OR LIMIT-TO ( EXACTKEYWORD , "Psychological Aspect" ) OR LIMIT-TO ( EXACTKEYWORD , "Psychosocial Care" ) OR LIMIT-TO ( EXACTKEYWORD , "COVID-19 Pandemic" ) OR LIMIT-TO ( EXACTKEYWORD , "Psychological Well-being" ) OR LIMIT-TO ( EXACTKEYWORD , "Quarantine" ) OR LIMIT-TO ( EXACTKEYWORD , "Disease Outbreaks" ) OR LIMIT-TO ( EXACTKEYWORD , "Hospitalization" ) OR LIMIT-TO ( EXACTKEYWORD , "Coronavirus" ) OR LIMIT-TO ( EXACTKEYWORD , "Health Program" ) OR LIMIT-TO ( EXACTKEYWORD , "Psychoeducation" ) OR LIMIT-TO ( EXACTKEYWORD , "Covid-19" ) OR LIMIT-TO ( EXACTKEYWORD , "Communicable Disease Control" ) OR LIMIT-TO ( EXACTKEYWORD , "Infection Prevention" ) OR LIMIT-TO ( EXACTKEYWORD , "Interpersonal Communication" ) OR LIMIT-TO ( EXACTKEYWORD , "Stress, Psychological" ) OR LIMIT-TO ( EXACTKEYWORD , "Emotion" ) OR LIMIT-TO ( EXACTKEYWORD , "Physiological Stress" ) OR LIMIT-TO ( EXACTKEYWORD , "Clinical Trial" ) OR LIMIT-TO ( EXACTKEYWORD , "Controlled Study" ) ) AND ( EXCLUDE ( AFFILCOUNTRY , "United States" ) OR EXCLUDE ( AFFILCOUNTRY , "United Kingdom" ) OR EXCLUDE ( AFFILCOUNTRY , "Italy" ) OR EXCLUDE ( AFFILCOUNTRY , "Canada" ) OR EXCLUDE ( AFFILCOUNTRY , "Australia" ) OR EXCLUDE ( AFFILCOUNTRY , "Germany" ) OR EXCLUDE ( AFFILCOUNTRY , "Undefined" ) OR EXCLUDE ( AFFILCOUNTRY , "Spain" ) OR EXCLUDE ( AFFILCOUNTRY , "Turkey" ) OR EXCLUDE ( AFFILCOUNTRY , "Netherlands" ) OR EXCLUDE ( AFFILCOUNTRY , "Switzerland" ) OR EXCLUDE ( AFFILCOUNTRY , "France" ) OR EXCLUDE ( AFFILCOUNTRY , "Poland" ) OR EXCLUDE ( AFFILCOUNTRY , "Sweden" ) OR EXCLUDE ( AFFILCOUNTRY , "Belgium" ) OR EXCLUDE ( AFFILCOUNTRY , "Saudi Arabia" ) OR EXCLUDE ( AFFILCOUNTRY , "Israel" ) OR EXCLUDE ( AFFILCOUNTRY , "Hong Kong" ) OR EXCLUDE ( AFFILCOUNTRY , "Japan" ) OR EXCLUDE ( AFFILCOUNTRY , "Ireland" ) OR EXCLUDE ( AFFILCOUNTRY , "South Korea" ) OR EXCLUDE ( AFFILCOUNTRY , "Malaysia" ) OR EXCLUDE ( AFFILCOUNTRY , "Portugal" ) OR EXCLUDE ( AFFILCOUNTRY , "Austria" ) OR EXCLUDE ( AFFILCOUNTRY , "Denmark" ) OR EXCLUDE ( AFFILCOUNTRY , "Singapore" ) OR EXCLUDE ( AFFILCOUNTRY , "Norway" ) OR EXCLUDE ( AFFILCOUNTRY , "Greece" ) OR EXCLUDE ( AFFILCOUNTRY , "Russian Federation" ) OR EXCLUDE ( AFFILCOUNTRY , "Finland" ) OR EXCLUDE ( AFFILCOUNTRY , "United Arab Emirates" ) OR EXCLUDE ( AFFILCOUNTRY , "New Zealand" ) OR EXCLUDE ( AFFILCOUNTRY , "Croatia" ) OR EXCLUDE ( AFFILCOUNTRY , "Ukraine" ) OR EXCLUDE ( AFFILCOUNTRY , "Argentina" ) OR EXCLUDE ( AFFILCOUNTRY , "Chile" ) OR EXCLUDE ( AFFILCOUNTRY , "Czech Republic" ) OR EXCLUDE ( AFFILCOUNTRY , "Qatar" ) OR EXCLUDE ( AFFILCOUNTRY , "Hungary" ) OR EXCLUDE ( AFFILCOUNTRY , "Slovenia" ) OR EXCLUDE ( AFFILCOUNTRY , "Puerto Rico" ) OR EXCLUDE ( AFFILCOUNTRY , "Slovakia" ) OR EXCLUDE ( AFFILCOUNTRY , "Oman" ) OR EXCLUDE ( AFFILCOUNTRY , "Macao" ) OR EXCLUDE ( AFFILCOUNTRY , "Luxembourg" ) OR EXCLUDE ( AFFILCOUNTRY , "Lithuania" ) OR EXCLUDE ( AFFILCOUNTRY , "Bosnia and Herzegovina" ) OR EXCLUDE ( AFFILCOUNTRY , "Latvia" ) OR EXCLUDE ( AFFILCOUNTRY , "Venezuela" ) OR EXCLUDE ( AFFILCOUNTRY , "Iceland" ) OR EXCLUDE ( AFFILCOUNTRY , "Uruguay" ) OR EXCLUDE ( AFFILCOUNTRY , "Georgia" ) OR EXCLUDE ( AFFILCOUNTRY , "Estonia" ) OR EXCLUDE ( AFFILCOUNTRY , "Dominican Republic" ) OR EXCLUDE ( AFFILCOUNTRY , "North Macedonia" ) OR EXCLUDE ( AFFILCOUNTRY , "Montenegro" ) OR EXCLUDE ( AFFILCOUNTRY , "Malta" ) OR EXCLUDE ( AFFILCOUNTRY , "Bulgaria" ) OR EXCLUDE ( AFFILCOUNTRY , "Tajikistan" ) OR EXCLUDE ( AFFILCOUNTRY , "Honduras" ) OR EXCLUDE ( AFFILCOUNTRY , "Bolivia" ) OR EXCLUDE ( AFFILCOUNTRY , "Albania" ) OR EXCLUDE ( AFFILCOUNTRY , "Uzbekistan" ) OR EXCLUDE ( AFFILCOUNTRY , "Moldova" ) OR EXCLUDE ( AFFILCOUNTRY , "Belarus" ) OR EXCLUDE ( AFFILCOUNTRY , "Armenia" ) )

**PsychINFO**

(outbreak* or epidemic* or pandemic* or epidemic* or coronavir* or "corona virus" or "corona pandemic" or covid19 or covid or "novel CoV" or sarscov2 or 2019ncov or "wuhan virus" or sars or "severe acute respiratory" or "coronavirus Infection" or coronavirus or mers-cov or "Middle East Respiratory Syndrome" or ebolavirus or ebola or "Ebola virus" or "Ebola Virus Disease" or "dengue virus" or "yellow fever virus" or "zika virus" or "Chikungunya Fever" or "Yellow fever" or measles).mp. [mp=title, abstract, heading word, table of contents, key concepts, original title, tests & measures, mesh word] AND ("psychological treatment" or "psychological intervention" or "psychological support" or "psychosocial treatment" or "psychosocial intervention" or "psychosocial support" or psychoeducation or counsel* or "cognitive behavioural therap" or "acceptance commitment therap" or "cognitive analytic therapy" or "family therapy" or "group therapy" or "problem solving therap" or "problem solving psychotherapy" or "mindfulness therapy" or "motivational interview" or "supportive psychotherapy" or "relaxation therapy" or "Psychodynamic psychotherapy" or "Imagery Psychotherapy" or "Rational-Emotive" or psychotherapy or "Brief Psychotherapy" or "Person Centered Psychotherapy" or "Interpersonal Psychotherapy" or "Cognitive Behavioral Therapy" or "Crisis Intervention").mp. [mp=title, abstract, heading word, table of contents, key concepts, original title, tests & measures, mesh word] AND exp Developing Countries/ or ((developing or less* developed or third world or under developed or middle income or low income or underserved or under served or deprived or poor*) adj1 (count* or nation? or state? or population?)).tw. or (lmic or lmics).tw. or exp Africa/ or exp Asia/ or exp South America/ or exp Latin America/ or exp Central America/ or (Africa or Asia or South America or Latin America or Central America).tw. or (Afghanistan* or Albania* or Algeria* or Samoa* or Angola* or Armenia* or Azerbaijan* or Bangladesh* or Bengali or Belarus* or Belize or Benin or Bhutan* or Bolivia* or Bosnia* or Herzegovina* or Botswana* or Brazil* or Bulgaria* or Burkina Faso or Burundi* or Cabo Verd* or Cape Verd* or Cambodia* or Cameroon* or Central African* or Chad* or China or Chinese or Colombia* or Comoros or Congo or Costa Rica* or Cote d'Ivoire or Ivory Coast or Cuba or Cuban or Djibouti or Dominica* or Ecuador or Egypt or El Salvador* or Eritrea* or Ethiopia* or Fiji* or Gabon* or Gambia* or Georgia* or Ghana* or Grenada* or Guatemala* or Guinea* or Guyan* or Haiti* or Hondura* or India or Indian* or Indonesia* or Iran* or Iraq* or Jamaica* or Jordan or Kazakh* or Kenya* or Kiribati or People* Republic of Korea or North Korea or Kosovo or Kosovar* or Kyrgyz* or Lao or Laos or Laotian* or Lebanon or Lebanes* or Lesotho or Liberia* or Libya* or Macedonia* or Madagascar* or Malawi* or Malaysia* or Maldives or Mali or Malian or Marshall Island* or Mauritania* or Mauriti* or Mexico or Mexican* or Micronesia* or Moldova* or Mongolia* or Montenegr* or Morocc* or Mozambique or Myanmar or Burmese* or Burma or Namibia* or Nepal* or Nicaragua* or Niger* or Pakistan* or Palau or Panama* or Paraguay* or Peru* or Philippin* or Romania* or Rwanda* or Samoa* or Sao Tome or Principe or Senegal* or Serbia* or Sierra Leone* or Solomon Island* or Somalia* or South Africa* or Sri Lanka or St Lucia or Saint Lucia or St Vincent or Saint Vincent or Grenadines or Sudan* or Suriname* or Swaziland* or Syria* or Tajik* or Tanzania* or Thai* or Timor* or Togo* or Tonga* or Tunisia* or Turkey or Turkish or Turkmen* or Tuvalu* or Uganda* or Ukrain* or Uzbeki* or Vanuatu* or Vietnam* or Viet nam* or West Bank or Gaza* or Palestin* or Yemen* or Zambia* or Zimbabw*).tw,sh,in.

**Cochrane trials**

(outbreak* OR epidemic* OR pandemic* OR epidemic* OR coronavir* OR "corona virus" OR "corona pandemic" OR covid19 OR covid OR "novel CoV" OR sarscov2 OR 2019ncov OR "wuhan virus" OR sars OR "severe acute respiratory" OR "coronavirus Infection" OR coronavirus OR mers-cov OR "Middle East Respiratory Syndrome" OR ebolavirus OR ebola OR "Ebola virus" OR "Ebola Virus Disease" OR "dengue virus" OR "yellow fever virus" OR "zika virus" OR "Chikungunya Fever" OR "Yellow fever" OR measles)

AND

("psychological treatment" OR "psychological intervention" OR "psychological support" OR "psychosocial treatment" OR "psychosocial intervention" OR "psychosocial support" OR psychoeducation OR counsel* OR "cognitive behavioural therap" OR "acceptance commitment therap" OR "cognitive analytic therapy" OR "family therapy" OR "group therapy" OR "problem solving therap" OR "problem solving psychotherapy" OR "mindfulness therapy" OR "motivational interview" OR "supportive psychotherapy" OR "relaxation therapy" OR "Psychodynamic psychotherapy" OR "Imagery Psychotherapy" OR "Rational-Emotive" OR psychotherapy OR "Brief Psychotherapy" OR "Person Centered Psychotherapy" OR "Interpersonal Psychotherapy" OR "Cognitive Behavioral Therapy" OR "Crisis Intervention")

AND

"Developing Countries" OR Africa OR Asia OR "South America" OR "Central America" OR "Latin America" OR developing OR "less developed" OR "third world" OR "under developed" OR "middle income" OR "low income" OR underserved OR "under served" OR deprived OR poor* OR Africa OR Asia OR "South America" OR "Central America" OR "Latin America" OR developing OR "less developed" OR "third world" OR "under developed" OR "middle income" OR "low income" OR Afghanistan* OR Albania* OR Algeria* OR Samoa* OR Angola* OR Armenia* OR Azerbaijan* OR Bangladesh* OR Bengali OR Belarus* OR Belize OR Benin OR Bhutan* OR Bolivia* OR Bosnia* OR Herzegovina* OR Botswana* OR Brazil* OR Bulgaria* OR "Burkina Faso" OR Burundi* OR "Cabo Verde" OR "Cape Verde" OR Cambodia* OR Cameroon* OR "Central African" OR Chad* OR China OR Chinese OR Colombia* OR Comoros OR Congo OR "Costa Rica" OR "Cote d Ivoire" OR "Ivory Coast" OR Cuba OR Cuban OR Djibouti OR Dominica* OR Ecuador OR Egypt OR "El Salvador" OR Eritrea* OR Ethiopia* OR Fiji* OR Gabon* OR Gambia* OR Georgia* OR Ghana* OR Grenada* OR Guatemala* OR Guinea* OR Guyan* OR Haiti* OR Hondura* OR India OR Indian* OR Indonesia* OR Iran* OR Iraq* OR Jamaica* OR Jordan* OR Kazakh* OR Kenya* OR Kiribati OR "People s Republic of Korea" OR "North Korea" OR Kosovo OR Kosovar* OR Kyrgyz* OR Lao OR Laos OR Laotian* OR Lebanon OR Lebanes* OR Lesotho OR Liberia* OR Libya* OR Macedonia* OR Madagascar* OR Malawi* OR Malaysia* OR Maldives OR Mali* OR "Marshall Islands" OR Mauritania* OR Mauriti* OR Mexico OR Mexican* OR Micronesia* OR Moldova* OR Mongolia* OR Montenegr* OR Morocc* OR Mozambique OR Myanmar OR Burmese* OR Burma OR Namibia* OR Nepal* OR Nicaragua* OR Niger* OR Pakistan* OR Palau OR Panama* OR Paraguay* OR Peru* OR Philippin* OR Romania* OR Rwanda* OR Samoa* OR Sao Tome OR Principe OR Senegal* OR Serbia* OR "Sierra Leone" OR Solomon Island* OR Somalia* OR "South Africa" OR "Sri Lanka" OR "St Lucia" OR "Saint Lucia" OR "St Vincent" OR "Saint Vincent" OR Grenadines OR Sudan* OR Suriname* OR Swaziland* OR Syria* OR Tajik* OR Tanzania* OR Thai* OR Timor* OR Togo* OR Tonga* OR Tunisia* OR Turkey OR Turkish OR Turkmen* OR Tuvalu* OR Uganda* OR Ukrain* OR Uzbeki* OR Vanuatu* OR Vietnam* OR "Viet nam" OR "West Bank" OR Gaza* OR Palestin* OR Yemen* OR Zambia* OR Zimbabw*
